# Dietary Inflammatory Index Positively Associated With High-Sensitivity C-Reactive Protein Level in Japanese From NIPPON DATA2010

**DOI:** 10.2188/jea.JE20180156

**Published:** 2020-02-05

**Authors:** Yunqing Yang, Atsushi Hozawa, Mana Kogure, Akira Narita, Takumi Hirata, Tomohiro Nakamura, Naho Tsuchiya, Naoki Nakaya, Toshiharu Ninomiya, Nagako Okuda, Aya Kadota, Takayoshi Ohkubo, Tomonori Okamura, Hirotsugu Ueshima, Akira Okayama, Katsuyuki Miura

**Affiliations:** 1 Division of Personalized Prevention and Epidemiology, Tohoku University Graduate School of Medicine, Sendai, Japan; 2 Department of Preventive Medicine and Epidemiology, Tohoku Medical Megabank Organization, Tohoku University, Sendai, Japan; 3 Center for Cohort Studies, Graduate School of Medical Sciences, Kyushu University, Fukuoka, Japan; 4 Department of Health and Nutrition, University of Human Arts and Sciences, Saitama, Japan; 5 Department of Public Health, Shiga University of Medical Science, Shiga, Japan; 6 Center for Epidemiologic Research in Asia, Shiga University of Medical Science, Shiga, Japan; 7 Department of Hygiene and Public Health, Teikyo University School of Medicine, Tokyo, Japan; 8 Department of Preventive Medicine and Public Health, Keio University School of Medicine, Tokyo, Japan; 9 Research Institute of Strategy for Prevention, Tokyo, Japan

**Keywords:** dietary inflammatory index, inflammation, CRP, Japanese, Japanese diet

## Abstract

**Background:**

It has been reported that chronic inflammation may play an important role in the pathogenesis of several serious diseases and could be modulated by diet. Recently, the Dietary Inflammatory Index (DII^®^) was developed to assess the inflammatory potential of the overall diet. The DII has been reported as relevant to various diseases but has not been validated in Japanese. Thus, in the present study, we analyzed the relationship between DII scores and high-sensitivity C-reactive protein (hs-CRP) levels in a Japanese population.

**Methods:**

Data of the National Integrated Project for Prospective Observation of Non-communicable Disease and its Trends in the Aged 2010 (NIPPON DATA2010), which contained 2,898 participants aged 20 years or older from the National Health and Nutrition Survey of Japan (NHNS2010), were analyzed. Nutrient intakes derived from 1-day semi-weighing dietary records were used to calculate DII scores. Energy was adjusted using the residual method. Levels of hs-CRP were evaluated using nephelometric immunoassay. Multiple linear regression analyses were performed.

**Results:**

After adjusting for age, sex, smoking status, BMI, and physical activity, a significant association was observed between DII scores and log(CRP+1) (standard regression coefficient = 0.05, *P* < 0.01). Although it was not statistically significant, the positive association was consistently observed in almost all age-sex subgroups and the non-smoker subgroup.

**Conclusions:**

The current study confirmed that DII score was positively associated with hs-CRP in Japanese.

## INTRODUCTION

Inflammation constitutes the body’s protective response to injury or infection and is generally beneficial to the body.^1^ However, when the inflammatory response proceeds disorderedly, acute inflammation can progress to chronic inflammation,^2^ which features sustained increased level of inflammatory cytokines, such as Interleukin 6 (IL-6), Tumor Necrosis Factor-α (TNF-α), and C-reactive protein (CRP). It has been reported that inflammation response and metabolic regulation are highly integrated and interdependent.^3^ Chronic inflammation, which is the dysfunction of the inflammatory response, can lead to a variety of diseases, such as diabetes, cancer, and depression, which seriously threatens health.^4^^–^^6^

Growing evidence has shown that diet plays a key role in the regulation of chronic inflammation. For example, the Mediterranean diet, which is rich in fish, monounsaturated fats from olive oil, fruits, vegetables, whole grains, and involves moderate alcohol consumption, has been shown to be associated with lower levels of inflammatory markers.^7^ In contrast, the Western diet, also known as the “obesogenic” diet, characterized by a high intake of saturated fat from red meat and dairy products, refined grains, and sugar, may promote metabolic disorders through pro-inflammatory mechanisms.^8^

Recently, a literature-derived, population-based diet quality assessing tool—the Dietary Inflammatory Index (DII^®^)—was developed for evaluating the inflammatory potential of one’s overall diet.^9^ The DII has been construct validated in American, European, Asian, and Australia individuals with inflammatory markers including CRP, IL-6, and TNF-α,^10^^–^^14^ and was reported to have associations with a variety of diseases. A recent published meta-analysis reported that there were consistent and significant positive associations between higher DII scores and cancer incidence and mortality across cancer types.^15^ Another review of cardiovascular diseases concluded that the DII was a useful tool for appraising the inflammatory potential of diet and for helping to explore the mechanisms between diet, inflammation, and cardio-metabolic diseases.^16^ A few relevant studies have been carried out in Asia; one of them was conducted in Japan.^17^

Japanese have enjoyed the world’s longest average life expectancy since 1985,^18^ which may partially be due to the Japanese traditional diet, *Washoku*, which was included in the United Nations Educational, Scientific and Cultural Organization list of Intangible Cultural Heritage in 2013.^19^^,^^20^ The Japanese diet incorporates high consumption of fish and soybean products and low consumption of animal fat and meat and has been reported as having a negative association with cardiovascular disease risks,^20^ psychological distress,^21^ and cancer.^22^ Whether the DII scores of the Japanese population that consumed a predominantly Japanese diet are applicable to epidemiological studies remains unclear. For this purpose, it was necessary to validate the DII using a Japanese database so that more researches could be conducted.

Therefore, we evaluated the association between DII scores and hs-CRP levels in Japanese using data from National Integrated Project for Prospective Observation of Non-Communicable Disease and Its Trends in the Aged 2010 (NIPPON DATA2010).^23^^–^^25^

## MATERIAL AND METHODS

### Study population

NIPPON DATA2010 was a nationally representative cohort study based on the National Health and Nutrition Survey of Japan in 2010 (NHNS2010),^26^ which used validated high-accuracy semi-weighing dietary records. The details of NHNS2010 and NIPPON DATA2010 have been described elsewhere.^26^^,^^27^ Briefly, 8,815 residents from 300 randomly selected survey areas throughout Japan participated in NHNS2010. Among them, 7,229 participants were aged 20 years or older, and 3,873 of the 7,229 completed the blood tests. Finally, 2,898 participants (1,239 men and 1,659 women, response rate: 74.6%) from the NHNS2010 agreed to be involved in the baseline survey of NIPPON DATA2010, which included electrocardiography, urinalysis, and questionnaires and was conducted in November 2010,^25^^,^^27^ and were subsequently recruited to the current study.

**Figure 1.**
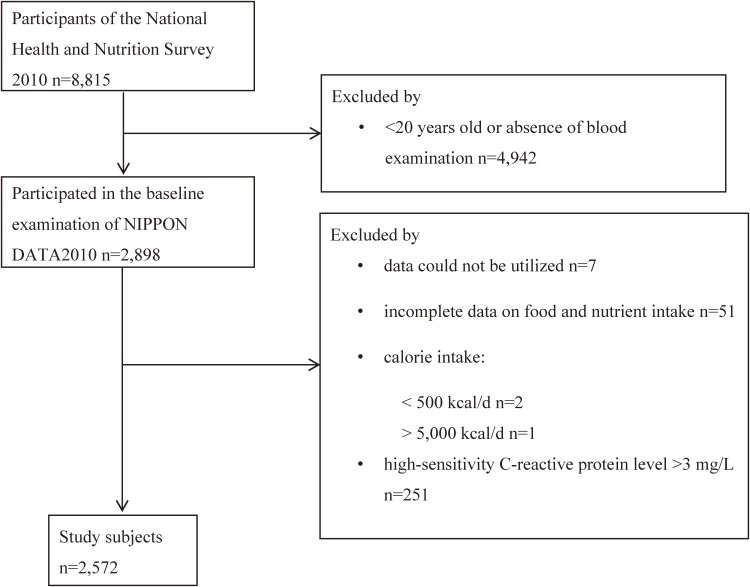
Flow diagram of study population. Participants were excluded for the following reasons: 1) younger than 20 years old or absence of blood examination; 2) without informed consent; 3) data could not be utilized; 4) having incomplete data on food and nutrient intake; 5) calorie intake less than 500 kcal/d or more than 5,000 kcal/d; 6) high-sensitivity C-reactive protein level >3 mg/L; 7) physical activity unknown; 8) smoking status unknown; 9) BMI could not be calculated.

Among the 2,898 participants, 7 participants could not be included due to unusable data, and 94 were excluded for the following reasons: incomplete data of food and nutrient intake (*n* = 51), extreme calorie intake <500 kcal/d (*n* = 2) or >5,000 kcal/d (*n* = 1)^28^; and missing data on weight or height (*n* = 2), physical activity (*n* = 4), or smoking status (*n* = 8). Considering the extremely low level of hs-CRP in Japanese, which is approximately one third of the median value in Caucasians,^29^^,^^30^ and the findings of one study conducted in six Asian cities suggesting that the reference CRP interval of Japanese was from 0.04 mg/L to 2.26 mg/L,^31^ we excluded participants with a CRP level >3 mg/L from the analyses (*n* = 251). Finally, a total of 2,572 participants were included in the analysis (Figure 1).

The Institutional Review Board of Shiga University of Medical Science approved this study (No. 22-29, 2010).

### Dietary intake and DII

Data on dietary intake were collected from 1-day semi-weighing household dietary records. Participants were asked to weigh and record all portions of foods, beverages, and nutrient supplements consumed by each household member in a whole day. In addition, participants were asked to carry out the dietary records on a normal day for representing dietary habits. Trained dietitians visited the participants’ homes to assist with and confirm the dietary records. Nutrient intakes were estimated using the Standard Tables of Food Composition in Japan, Fifth Revised and Enlarged Edition.^26^^,^^32^

The DII was developed as a diet quality-assessing tool based on the inflammatory potential of the overall diet. Forty-five food or nutrient parameters were identified by their effects on six inflammatory markers (IL-1β, IL-4, IL-6, IL-10, TNF-α, and CRP), and a global standard database was created for comparing DII scores in diverse populations. A more detailed description of the DII has been provided elsewhere.^9^ Briefly, the DII provided an overall inflammatory effect score, a global daily mean intake, and a standard deviation for each food parameter. First, every nutrient intake was transformed to a Z-score using the standard values described above. To minimize the ‘right skewing,’ each Z-score was converted to a percentile value, which was then doubled, and 1 was subtracted from the doubled percentile value. Next, the centered value was multiplied by its respective overall inflammatory effect score. Finally, all parameter-specific DII scores were summed to achieve the overall DII score for each subject.

In the current study, 26 food or nutrient parameters, including vitamin B12, carbohydrate, cholesterol, total fat, iron (Fe), protein, saturated fat, magnesium (Mg), zinc (Zn), vitamin A, β-carotene, vitamin D, vitamin E, thiamine, riboflavin, niacin, vitamin B6, folic acid, vitamin C, monounsaturated fatty acid (MUFA), polyunsaturated fatty acid (PUFA), fiber, n-3 fatty acid, n-6 fatty acid, alcohol, and onion could be used to calculate DII scores (eTable 1). Among these, alcohol consumption was calculated from data of lifestyle surveys, and the others were derived from dietary records. Energy adjustment was performed using the residual method.^33^

### C-reactive protein

Fasting blood samples were drawn from all participates in November 2010. Levels of hs-CRP were measured using nephelometric immunoassay at a commercial laboratory (SRL, Tokyo, Japan).

### Covariates

Anthropometric measurements were performed by trained staff. Height and weight were measured and used to calculate the BMI as the ratio of weight to the square of height. Lifestyle surveys, including information on smoking (current, former, or never smoker), physical activity (Metabolic equivalents [METs]/d) and antilipidemic agent use (user or non-user), were conducted by public health nurses using a standard questionnaire.^26^ Information on socioeconomic status, such as marital status (married or unmarried), education (junior high school and below, high school, or university and above), and equivalent household expenditure was collected from the self-administered questionnaires. (1 Yen = 0.008989 United States dollar as of January 2018)

### Statistical analysis

The characteristics of participants and food intakes across the DII quartiles were compared using chi-square test for categorical variables and ANOVA for continuous variables. Levels of hs-CRP were log-transformed due to its right-skewed distribution. To determine the association between DII scores and log-transformed (hs-CRP+1) [log(CRP+1)], Spearman’s correlation and multiple linear regression were analyzed. As potential confounders, age, sex, smoking status, BMI, and physical activity were adjusted. Moreover, analyses were further stratified by sex (men and women), age group (aged <45, 45–54, 55–64, 65–74, and ≥75 years) and smoking status (never-smoker, former-smoker, and current smoker). Additionally, we analyzed other factors as covariates, including economic status, marital status, education, and antilipidemic agent use. All statistical analyses were performed using Statistical Analysis Systems statistical software package version 9.4 (SAS Institute, Cary, NC, USA).

## RESULTS

The mean DII score of the study participants was 0.82, with a SD of 1.75. Table 1 showed the characteristics of the study participants across DII score quartiles: −5.04 ≤ Q1 < −0.38; −0.38 ≤ Q2 < 0.91; 0.91 ≤ Q3 < 2.18; 2.18 ≤ Q4 ≤ 4.94. The proportion of women decreased with DII score quartiles, indicating that, compared with men, women consumed a more anti-inflammatory diet. Participants in Q4, the most pro-inflammatory diet-consuming group, were more likely to be younger, antilipidemic agent non-user, underweight or overweight, smokers, with higher physical activity, lower equivalent household expenditure, and more likely to be single than participants in other quartiles.

**Table 1.** Characteristics across quartiles of Dietary Inflammatory Index (DII^®^) scores

	DII quartiles^a^								

	Q1		Q2		Q3		Q4		
Characteristics	*n*	%	*n*	%	*n*	%	*n*	%	*P*-value
**Median DII score**	−1.38		0.33		1.55		2.85		<0.01
**Sex**									
** Men**	239	37.2	253	39.4	274	42.6	320	49.8	<0.01
** Women**	404	62.8	390	60.7	369	57.4	323	50.2	
**Age, years, mean (SD)**	64.4 (12.3)		61.3 (14.8)		56.1 (16.5)		52.3 (16.5)		<0.01
**BMI, kg/m^2^**									
** <18.5**	38	5.9	42	6.5	32	5.0	55	8.6	0.03
** 18.5 to <25.0**	448	69.7	441	68.6	430	66.9	400	62.2	
** ≥25.0**	157	24.4	160	24.9	181	28.2	188	29.2	
**Smoking**									
** Current smoker**	51	7.9	71	11.0	110	17.1	162	25.2	<0.01
** Former smoker**	111	17.3	131	20.4	124	19.3	132	20.5	
** Never-smoker**	481	74.8	441	68.6	409	63.6	349	54.3	
**Physical activity, METs/d**	37.3 (8.0)		37.0 (7.9)		37.5 (9.0)		38.6 (9.6)		<0.01
**Antilipidemic agent^b^**									
** User**	126	19.6	115	17.9	87	13.6	63	9.8	<0.01
** Non-user**	517	80.4	528	82.1	555	86.5	580	90.2	
**Marital status^b^**									
** Married**	513	80.0	511	79.6	483	75.4	456	71.6	<0.01
** Single**	128	20.0	131	20.4	158	24.7	181	28.4	
**Education^b^**									
** Middle or lower**	167	26.0	162	25.2	145	22.6	142	22.1	0.47
** High school**	279	43.4	267	41.5	296	46.1	288	44.9	
** University or higher**	197	30.6	214	33.3	201	31.3	212	33.0	
**Equivalent household expenditure, million Yen/month, mean (SD)^b^**									
	16. (10.1)		15.7 (14.5)		14.6 (12.6)		14.3 (18.7)		<0.01

BMI, body mass index; METs, metabolic equivalents; SD, standard deviation.

^a^DII quartiles: −5.04 ≤ Q1 < −0.38; −0.38 ≤ Q2 < 0.91; 0.91 ≤ Q3 < 2.18; 2.18 ≤ Q4 ≤ 4.94.

^b^Sample size: antilipidemic agent use = 2,571; marital status = 2,561; education = 2,570; equivalent household expenditure = 2,380.

Comparing the food intakes distribution across the DII quartiles, we found certain food intakes were related to the decrease or increase of DII scores. With the increase in cereal, meat, fat, and oil intake, the DII score increased. On the other hand, potato, bean, nut and seed, vegetable, fruit, mushroom, seaweed, seafood, milk, and nutrients supplementary food showed an effect of lowering DII score in the current study (Table 2).

**Table 2.** Food intakes across quartiles of Dietary Inflammatory Index (DII^®^) scores^a^

Food item (g)	Q1	SD	Q2	SD	Q3	SD	Q4	SD
Cereal	393.28	145.20	425.79	156.73	449.87	167.15	507.25	191.50
Potato	80.32	84.22	61.00	68.13	52.05	62.93	41.73	53.20
Sugar and Sweeteners	7.89	8.65	7.31	7.88	6.96	8.81	7.77	11.46
Bean	99.18	90.08	75.14	81.23	53.39	64.72	41.92	59.39
Nut and seed	4.45	10.23	2.98	10.01	1.78	6.84	1.24	5.06
Vegetable	459.00	179.31	329.42	145.18	258.89	134.71	179.58	111.20
Fruit	190.88	150.37	138.68	129.31	98.97	113.66	61.13	93.60
Mushrooms	28.83	35.24	22.05	29.48	15.68	24.44	11.57	20.95
Seaweeds	19.13	31.83	11.94	20.51	11.31	20.83	8.04	16.82
Seafood	107.93	76.29	89.63	71.39	79.91	77.64	57.26	66.25
Meat	63.20	57.03	68.38	59.69	78.28	67.28	92.12	80.79
Egg	33.69	30.66	37.99	32.36	36.65	34.39	34.73	33.49
Milk	118.36	122.92	111.36	127.04	100.23	132.26	93.13	125.75
Fat and oil	8.92	8.44	8.95	8.44	10.31	9.73	10.58	8.83
Confectionery	19.86	34.24	26.35	43.89	26.04	44.60	36.36	56.19
Preferred beverage	766.11	469.28	720.00	471.81	702.66	511.27	720.13	522.49
Seasoning and Spice	99.87	146.78	92.96	81.50	84.59	78.21	91.88	95.38
Nutrients supplementary food	19.03	58.99	18.39	61.35	18.22	69.16	10.63	62.21

SD, standard deviation.

^a^DII quartiles: −5.04 ≤ Q1 < −0.38; −0.38 ≤ Q2 < 0.91; 0.91 ≤ Q3 < 2.18; 2.18 ≤ Q4 ≤ 4.94.

We did not observe significant correlation between DII scores and log(CRP+1) when analyzing in crude (*r* = 0.02, *P* = 0.41). After adjusting for age, sex, smoking status, BMI, and physical activity, a significant relationship was observed between DII scores and log(CRP+1) (standard regression coefficient of total = 0.05, *P* < 0.01) (Table 3). The standardized regression coefficient of the covariates was reduced in the order of BMI (0.33), age (0.14), current smoking (0.06), physical activity (0.06), and DII score (0.05).

**Table 3.** Multiple linear regression analysis between log-transformed hs-CRP and other variables, stratified by sex^a^

	Men *n* = 1,086^c^					Women *n* = 1,486^c^					Total *n* = 2,572^d^				
Variable	Standardized β	β	95% CI		*P*	Standardized β	β	95% CI		*P*	Standardized β	β	95% CI		*P*
DII score^b^	0.05	0.01	−0.003	0.02	0.14	0.06	0.01	0.001	0.02	0.02	0.05	0.01	0.003	0.02	<0.01
Age^b^	0.13	0.003	0.001	0.004	<0.01	0.13	0.002	0.002	0.003	<0.01	0.14	0.003	0.002	0.003	<0.01
BMI^b^	0.27	0.03	0.02	0.03	<0.01	0.37	0.03	0.03	0.04	<0.01	0.33	0.03	0.026	0.032	<0.01
Sex (ref. women)											0.007	0.004	−0.02	0.03	0.76
Smoking (ref. never-smokers)															
Former smokers	0.03	0.02	−0.02	0.06	0.30	0.04	0.05	−0.01	0.11	0.07	0.03	0.02	−0.01	0.05	0.17
Current smokers	0.10	0.07	0.03	0.12	<0.01	−0.001	−0.001	−0.06	0.06	0.97	0.06	0.05	0.02	0.09	<0.01
Physical activity^b^	−0.06	−0.002	−0.003	−0.00001	0.06	−0.06	−0.003	−0.005	−0.001	0.01	−0.06	−0.002	−0.003	−0.001	<0.01

BMI, body mass index; CI, confidence interval; hs-CRP, highly sensitive C-reactive protein.

^a^hs-CRP, high-sensitivity C-reactive protein; DII, Dietary inflammatory index; energy was adjusted by residual method.

^b^Continuous variable.

^c^Adjusted for age, BMI, smoking status, and physical activity.

^d^Adjusted for age, BMI, sex, smoking status, and physical activity.

Furthermore, the results of multiple linear regression analysis stratified by sex and age group are shown in Table 4. Consistent positive associations were observed both in men (although it was not statistically significant, standardized regression coefficient = 0.05, *P* = 0.14) and women (standardized regression coefficient = 0.06, *P* = 0.02). All age groups displayed a positive association, (standardized regression coefficient_<45_ = 0.05, standardized regression coefficient_45–54_ = 0.03, standardized regression coefficient_55–64_ = 0.03, standardized regression coefficient_65–74_ = 0.05, standardized regression coefficient_≥75_ = 0.10). The highest standardized regression coefficient between the DII and log(CRP+1) was observed in the ≥75 years age group. As regards age-sex combined subgroups, all subgroups except for men aged <45 years and women aged 55–64 years showed positive relationships between DII scores and log(CRP+1).

**Table 4.** Multiple linear regression analysis between log-transformed hs-CRP and Dietary Inflammatory Index (DII^®^) scores, stratified by age and sex

		Men			Women			Total	
Age, years	*N*	standardized β	*P*^a^	*N*	standardized β	*P*^a^	*N*	standardized β	*P*^b^
<45	212	−0.05	0.42	361	0.11	0.02	573	0.05	0.21
45–54	135	0.05	0.53	202	0.02	0.75	337	0.03	0.51
55–64	255	0.10	0.12	336	−0.04	0.50	591	0.03	0.43
65–74	309	0.01	0.91	369	0.08	0.11	678	0.05	0.19
≥75	175	0.04	0.61	218	0.14	0.04	393	0.10	0.05
Total	1,086	0.05	0.14	1,486	0.06	0.02	2,572	0.05	<0.01

hs-CRP, high-sensitivity C-reactive protein.

^a^Adjusted for age, smoking status, BMI, and physical activity.

^b^Adjusted for age, sex, smoking status, BMI, and physical activity.

Additionally, we analyzed other factors as covariates, including economic status, marital status, education, and antilipidemic agent use, gaining unchanging result (standardized regression coefficient = 0.06, *P* < 0.01). Further, the positive association was observed in the never-smoker (standardized regression coefficient = 0.06, *P* = 0.01, *n* = 1,680) and former-smoker (standardized regression coefficient = 0.08, *P* = 0.07, *n* = 498) subgroup, but not in the current-smoker subgroup (standardized regression coefficient = −0.02, *P* = 0.71, *n* = 394), when analysis was stratified by smoking status.

## DISCUSSION

In our cross-sectional study, we observed a positive association between DII scores and hs-CRP levels in participants of NIPPON DATA2010. The findings were consistent across almost all age-sex subgroups. The results suggested that the DII was applicable to the Japanese population.

### Previous studies on DII scores and CRP levels

To the best of our knowledge, there have been 21 previous studies that investigated the association between DII scores and CRP levels (Table 5). Fourteen of them concurred with our conclusion that the DII scores positively associated with CRP levels. Of the other seven studies, five concluded that the DII score was associated with other inflammatory markers. To our best knowledge, ours is the first written report to correctly validate the DII in Japanese with CRP.

**Table 5.** Previous research on association between Dietary Inflammatory Index (DII^®^) and CRP

Author	Year	Country or race	Number of food parameters	Inflammatory markers	Risk estimate
**Vahid F**^47^	2018	Iran	31	TNF-α^a^ IL-4^a^ IL-10^a^ IL-1β^a^ CRP^a^ IL-6^a^	Partial correlation coefficient
					CRP (mg/L) 0.328 *P* > 0.001
					TNF-α (pg/ml) 0.373 *P* > 0.001
					IL-6 (pg/ml) 0.337 *P* > 0.001
					IL-1β (pg/ml) 0.326 *P* > 0.001
					IL-4 (pg/ml) 0.046 *P* = 0.544
					IL-10 (pg/ml) −0.333 *P* > 0.001

**Phillips CM**^48^	2018	Ireland	26	Inflammatory score C3^a^ CRP IL-6 TNF-α Adiponectin Leptin Resistin WBC^a^ Neutrophils Lymphocytes Monocytes Eosinophils Basophils Neutrophil to lymphocyte ratio	Mean of < Median E-DII vs > Median E-DII Inflammatory score 7.74 ± 0.12 vs 8.29 ± 0.10 *P* < 0.001 C3 (mg/dL) 134.31 ± 0.78 vs 136.90 ± 0.76 *P* = 0.04 CRP (mg/L) 2.19 ± 0.12 vs 2.45 ± 0.11 *P* = 0.03 IL-6 (pg/mL) 2.72 ± 0.14 vs 3.02 ± 0.15 *P* < 0.001 TNF-α (pg/mL) 6.23 ± 0.08 vs 6.51 ± 0.09 *P* = 0.001 Adiponectin (ng/mL) 6.05 ± 0.13 vs 5.41 ± 0.13 *P* < 0.001 Leptin (ng/mL) 2.85 ± 0.12 vs 2.78 ± 0.10 *P* = 0.11 Resistin (ng/mL) 5.64 ± 0.10 vs 5.78 ± 0.11 *P* = 0.50 WBC (109/L) 5.85 ± 0.07 vs 6.14 ± 0.06 *P* = 0.001 Neutrophils (109/L) 3.23 ± 0.04 vs 3.48 ± 0.04 *P* = <0.001 Lymphocytes (109/L) 1.83 ± 0.02 vs 1.86 ± 0.03 *P* = 0.37 Monocytes (109/L) 0.51 ± 0.005 vs 0.54 ± 0.01 *P* < 0.001 Eosinophils (109/L) 0.20 ± 0.004 vs 0.21 ± 0.005 *P* = 0.06 Basophils (109/L) 0.031 ± 0.001 vs 0.033 ± 0.001 *P* = 0.03 Neutrophil to lymphocyte ratio 1.89 ± 0.03 vs 2.04 ± 0.03 *P* < 0.001

**Shivappa N**^49^	2018	USA	26	CRP	OR (95%CI) DII continuous (age adjusted) 1.13 (1.07, 1.20) DII continuous (multivariable) 1.12 (1.05, 1.19)

**Shivappa N**^45^	2018	Germany	Not found	CRP	*r* = 0.12

**Farhangi MA**^50^	2018	Iran	28	CRP IL-6	Beta estimate (95%CI) for the association Q_4_vsQ_1_ Men 0.97 (0.89, 1.06) ​ Women 0.93 (0.67, 1.30)

**Almeida-de-Souza J**^51^	2017	Portugal	31	CRP IL-6 C3 C4^a^ Overall score	OR (95%CI) T_3_vsT_1_ CRP^a^ 2.33 (0.88, 6.20) ​ IL-6 3.38 (1.24, 9.20) ​ C3^a^ 1.71 (0.63, 4.66) ​ C4^a^ 3.12 (1.21, 8.10) ​ Overall 5.61 (2.00, 15.78)

**Tabung FK**^52^	2017	USA	38	CRP IL-6 TNFαR2^a^ Adiponectin	Percentage change (95%CI) Q_5_vsQ_1_ NHS-II^a^: CRP^a^ +49% (+25%, +77%) ​ IL-6 +21% (+9%, +33%) ​ TNFαR2 +4% (+1%, +8%) ​ Adiponectin −10% (−10%, −4%) Q_5_vsQ_1_ HPFS^a^: CRP^a^ +29% (+15%, +44%) ​ IL-6 +24% (+12%, +38%) ​ TNFαR2 +5% (+1%, +8%) ​ Adiponectin −4% (−9%, +2%)

**Wirth MD**^11^	2017	African Americans	31	CRP IL-6	Percentile regression (95%CI) CRP^a^ β 0.75: 3.95 (1.71, 6.19) ​ β 0.90: 6.83 (1.11, 12.55)

**Vahid F**^13^	2017	Iran	31	CRP IL-6	Beta estimates (95%CI) CRP^a^ 0.04 (−0.09, 0.18) IL-6 0.16 (0.02, 0.30)

**Julia C**^53^	2017	France	36	CRP	OR (95%CI) T_3_vsT_1_ 1.32 (0.89, 1.95)

**Shivappa N**^54^	2017	European	25	CRP TNF-α IL-6, 1,2,4,10, IFN-γ^a^ sICAM^a^ sVCAM^a^	Beta estimates (95%CI) T_3_vsT_1_ CRP^a^ 0.09 (−0.18, 0.36) ​ TNF-α 0.13 (0.007, 0.26) ​ IL-6 0.09 (−0.22, 0.40) ​ IL-1 0.30 (0.02, 0.58) ​ IL-2 0.42 (0.04, 0.79) ​ IL-4 0.17 (−0.25, 0.59) ​ IL-10 0.09 (−0.17, 0.35) ​ INF-γ 0.58 (0.09, 1.06) ​ ICAM^a^ 0.02 (−0.08, 0.11) ​ VCAM^a^ 0.07 (0.01, 0.13)

**Shivappa N**^55^	2017	USA	27	CRP	OR (95%CI) Q_4_vsQ_1_ 1.53 (1.20, 1.95)

**Bodén S**^56^	2017	Sweden	30	CRP IL-6	Beta coefficients (95%CI) Q_4_vsQ_1_ CRP^a^ 0.41 (0.16, 0.67) ​ IL-6 0.26 (0.06, 0.46)

**Kizil M**^57^	2016	Turkey	25	CRP	*r* = 0.35

**Sarbattama Sen**^58^	2016	USA	28	CRP WBC	Beta coefficients (95%CI) CRP^a^ Continuous 0.08 (0.02, 0.14) ​ Q_4_vsQ_1_ 0.25 (−0.01, 0.50) WBC^a^ Continuous −0.03 (−0.11, 0.05) ​ Q_4_vsQ_1_ −0.14 (−0.45, 0.17)

**Akbaraly T**^37^	2016	UK	27	CRP IL-6	CRP^a^ T1 −0.13 ± 1.3 ​ T2 0.02 ± 1.3 ​ T3 0.03 ± 1.3 IL-6 T1 −0.12 ± 1.3 ​ T2 0.002 ± 1.3 ​ T3 0.04 ± 1.3

**Tabung FK**^12^	2015	USA	32	IL-6 CRP TNFα-R2 Overall score	Beta coefficients (95%CI) Q_5_vsQ_1_ IL-6 1.26 (1.15, 1.38) ​ CRP^a^ 1.07 (0.95, 1.2) ​ TNFα-R2 81.43 (19.15, 143.71) ​ Overall 0.26 (0.12, 0.40) OR Q_5_vsQ_1_ (95%CI) CRP^a^ NSAIDs^a^ non-user 1.67 (1.09, 2.55) ​ NSAIDs^a^ user 0.99 (0.65, 1.52)

**Shivappa N**^10^	2015	Belgians	17	CRP Leucocyte count Fibrinogen Homocysteine IL-6	OR (95%CI) CRP^a^ 1.03 (0.86, 1.17) IL-6 1.19 (1.04, 1.36) Homocysteine 1.56 (1.25, 1.94) Fibrinogen 1.08 (0.78, 1.48)

**Alkerwi A**^59^	2014	Luxembourg	24	CRP	*P* for trend = 0.39

**Wirth MD**^60^	2014	USA	Not found	CRP IL-6 TNF-α	OR (95%CI) Q_4_vQ_1_ 1.57 (0.85, 2.88)

**Shivappa N**^34^	2013	USA	44 (24-hour dietary recalls) 28 (7-day dietary recalls)	CRP	OR (95%CI) T_3_vsT_1_ 24 hour dietary recalls: 1.47 (1.03, 2.12) ​ 7-day dietary recall: 1.61 (1.15, 2.27)

C3, complement C3; C4, complement C4; CRP, C-reactive protein; DII, Dietary inflammatory index; HPFS, Health Professionals Follow-Up Study; IL, Interleukin; NHS-II, Nurses’ Health Study II; NSAIDs, non-steroidal anti-inflammatory drugs; sICAM, soluble intercellular cell adhesion molecule; sVCAM, soluble vascular cell adhesion molecule; TNF-α, Tumor Necrosis Factor-α; TNF-αR2, Tumor Necrosis Factor-α Receptor 2; WBC, white blood cell.

In the current study, 18 items of 45 food parameters were unavailable for DII score calculation, which were caffeine, eugenol, garlic, ginger, saffron, selenium, trans fat, turmeric, green/black tea, flavan-3-ol, flavones, flavonols, flavonones, anthocyanidins, isoflavones, pepper, thyme/oregano, and rosemary. However, in previous studies, the number of food parameters used was between 17 and 44. Furthermore, a construct validation study using two different diet record methods, 24-hour dietary recalls and 7-day dietary recalls, reported that the reduction of available food parameters would not lead to a large drop-off in the predictive ability of DII.^34^ Thus, the 26 food parameters we used might be sufficient for validation.

### International comparison of DII scores

The mean DII score of this study’s participants was 0.82 (standard deviation, 1.75). The Japanese diet is characterized by lower fat intake and higher soy and fish consumption.^35^ Therefore, we expected that the mean DII score in our study would be lower than that reported for western populations. However, our results did not bear out this expectation. For instance, a study on the association between the DII score and memory function using a population-based national sample of elderly Americans reported a mean DII score of −0.25 (standard error, 0.07).^36^ The mean DII score of the Whitehall II study, which was carried out in the United Kingdom, was −0.03 (standard deviation, 1.3).^37^ We reasoned that it may be due to the different food parameters used. Although DII score is calculated based on the global standard database, it cannot be used to compare the inflammatory potential of diets of different countries directly without using a unified set of food parameters.

### Factors relevant to elevated CRP levels

The multiple linear regression analysis suggested that ageing, smoking, and being overweight were positively associated with CRP levels, while physical activity was inversely related. We could not determine the causality through the cross-sectional studies; however, it is unlikely that an increased CRP level leads to smoking. Moreover, many previous studies reported similar results that CRP levels were higher among current smokers.^38^^–^^40^ According to our analysis, the effect of smoking on CRP levels was similar to the effect of DII scores (standardized regression coefficient = 0.06, *P* < 0.01).

BMI and physical activity had inverse effects on CRP levels. Our results are in accordance with several previous studies. A systematic review and a reciprocal Mendelian randomization study suggested that obesity was correlated with elevated levels of CRP.^41^^,^^42^ Moreover, increasing evidence points to the negative association between physical activity and inflammatory biomarker levels.^43^^,^^44^ Given the health benefits in metabolic regulation from physical activity, we propose that, besides diet, weight control, smoking cessation, and increasing physical activity may contribute to lower CRP levels.

We found a positive association between DII scores and CRP levels in almost all age-sex subgroups, but not in a few young men and women aged 55–64 years. This was likely due to that, in the current study, participants in the youngest male subgroup had the highest smoking rate (40.09% current smoker and 22.17% former smoker). According to previous researches, smoking was an important confounder due to its relatively strong inflammatory effect. The strong inflammatory effect might cover the effect bought by diet.^45^ As described in the results, only the current-smoker subgroup did not show the positive association. The smaller sample size of current-smoker may be partially responsible; however, we still believed that smoking could be considered as a reason of the negative association in this subgroup of young men. Moreover, women in the 55–64 year age group were possibly in menopause, which has been confirmed to associate with increases in CRP levels.^46^ The effect of menopause might modify the association between DII scores and CRP levels. Further study investigating DII scores and CRP levels in this age-sex group might be required.

### Strengths and limitations

Our study has several strengths. To our best knowledge, this is the first study of the inflammatory potential of the world-renowned Japanese diet and validation of the DII among Japanese. In addition, the participants of NIPPON DATA2010 were collected from all over Japan, with a large age span, ensuring a good representation of the Japanese population. This allowed the relatively detailed analysis of the association between DII scores and CRP levels in different sex and age groups.

Certain limitations should be mentioned. It was difficult to infer the temporal association between DII scores and CRP levels with the cross-sectional study design. However, it was almost impossible that participants changed their diets due to a high CRP level. Another limitation was the lack of information on anti-inflammatory medication use. The effect of diet on inflammation might partially be masked by using medicine^12^ that could lead to underestimation, which might partially explain why only weak associations were observed in the current study. Future studies should stratify analysis of the association between DII and CRP by anti-inflammatory medication.

In conclusion, we confirmed that a positive association between DII scores and CRP levels was observed in the Japanese population. The findings were consistent for almost all age-sex subgroups and the never-smoker subgroup.

## APPENDIX A. SUPPLEMENTARY DATA

eTable 1. Food parameters of Dietary Inflammatory Index (DII^®^) used in the current study
