# Supplementary material for: Dietary Inflammatory Index Positively Associated With High-Sensitivity C-Reactive Protein Level in Japanese From NIPPON DATA2010
Source: J Epidemiol. 2020 Feb 5;30(2):98–107. doi: 10.2188/jea.JE20180156 (PMC6949183; doi:10.2188/jea.JE20180156)
Supplement: Supplementary file 1 [file je-30-098-s001.pdf]

eTable 1. Food parameters of Dietary Inflammatory Index (DII®) used in the current study

**Food parameter used**[1]	**Overall inflammatory effect score**[1]	**Global daily mean intake, units/d**[1]	**SD**[1]	**NIPPON DATA2010 mean intake, units/d**[a]	**Mean DII score of food parameter**[b]
**Alcohol, g**	-0.278	13.98	3.72	10.12	0.150
**Vitamin B12, µg**	0.106	5.15	2.70	6.65	0.001
**Vitamin B6, mg**	-0.365	1.47	0.74	2.40	0.060
**β-Carotene, µg**	-0.584	3718	1720	4465.06	-0.032
**Carbohydrate, g**	0.097	272.2	40.0	270.15	-0.001
**Cholesterol, mg**	0.110	279.4	51.2	315.06	0.013
**Total fat, g**	0.298	71.4	19.4	53.68	-0.159
**Fiber, g**	-0.663	18.8	4.9	16.07	0.217
**Folic acid**	-0.190	273.0	70.7	323.48	-0.039
**Fe, mg**	0.032	13.35	3.71	8.69	-0.023
**Mg, mg**	-0.484	310.1	139.4	264.49	0.116
**MUFA, g**	-0.009	27.0	6.1	18.12	0.006
**Niacin, mg**	-0.246	25.9	11.77	15.56	0.141
**n-3 Fatty acids, g**	-0.436	1.06	1.06	2.42	-0.247
**n-6 Fatty acids, g**	-0.159	10.80	7.50	9.54	0.021
**Onion, g**	-0.301	35.9	18.4	28.99	0.094
**Protein, g**	0.021	79.4	13.9	70.88	-0.007
**PUFA, g**	-0.337	13.88	3.76	12.02	0.100
**Riboflavin, mg**	-0.068	1.70	0.79	1.70	0.023

**Saturated fat, g**	0.373	28.6	8.0	14.28	-0.320
**Thiamin, mg**	-0.098	1.70	0.66	2.04	0.062
**Vitamin A, RE**	-0.401	983.9	518.6	561.48	0.221
**Vitamin C, mg**	-0.424	118.2	43.46	131.17	0.067
**Vitamin D, µg**	-0.446	6.26	2.21	8.51	0.027
**Vitamin E, mg**	-0.419	8.73	1.49	9.75	0.183
**Zn, mg**	-0.313	9.84	2.19	8.16	0.150

MUFA, mono-unsaturated fatty acid; PUFA, poly-unsaturated fatty acid; RE, retinol equivalent; SD, standard deviation.

[a]Energy-adjusted nutrient intakes calculated using the residual method

[b]DII score of food parameter = ((NIPPON DATA2010 mean intake-Global daily mean intake)/SD*2-1)*Overall inflammatory effect score

1. Shivappa N, Steck SE, Hurley TG, Hussey JR, Hebert JR. Designing and developing a literature-derived, population-based dietary inflammatory index. *Public Health Nutr.* 2014;17(8):1689-1696.
